# Validation of the Penn State Worry Questionnaire in a sample of Afghan students

**DOI:** 10.3389/fpsyg.2026.1800125

**Published:** 2026-05-28

**Authors:** Mohammad Sajjad Afsharzada, Sajjad Saadat, Basir Ahmad Azizi, Wahidh Talbian

**Affiliations:** 1 Department of Education, Psychology and Social Work, Faculty of Human Sciences, Mid Sweden University, Östersund, Sweden; 2 Neuroscience Research Center, Guilan University of Medical Sciences, Rasht, Iran; 3 Department of Counseling, Faculty of Education, Herat University, Herat, Afghanistan

**Keywords:** Dari/Farsi version, Penn State Worry Questionnaire, reliability, validation, validity

## Abstract

**Background:**

Worry is a central cognitive feature of anxiety and emotional distress, and its accurate assessment is vital for understanding psychological well-being in high-stress societies such as Afghanistan.

**Objective:**

The present study aimed to translate and validate the Dari/Farsi version of the of Penn State Worry Questionnaire (PSWQ) in a sample of Afghan students. It employed a descriptive and validation-based approach.

**Method:**

The target population of the present research included 254 undergraduate students studying at Herat University, Afghanistan, during the 2024–2025 academic year. Participants were recruited through convenience sampling and responded to an online questionnaire distributed via Qualtrics. To assess temporal stability, a subsample of 40 participants completed the (PSWQ) again after a two-week interval. Data were collected using the PSWQ and the Depression, Anxiety, and Stress Scale (DASS-21).

**Results:**

Confirmatory factor analysis supported the unidimensional structure of the PSWQ, indicating an acceptable model fit (*χ^2^*/df = 2.58, CFI = 0.94, RMSEA = 0.07). The PSWQ scores demonstrated moderate positive correlations with depression, anxiety, and stress as measured by the DASS-21 (*r* = 0.53, 55, and 0.59, respectively; *p* < 0.05). In addition, the scale showed satisfactory test–retest reliability over 2 weeks, (*r* = 0.70, *p* < 0.05), and high internal consistency (*α* = 0.92).

**Conclusion:**

Overall, the findings confirm that the Dari/Farsi version of the PSWQ possesses robust psychometric characteristics. Therefore, it can be confidently utilized as a reliable instrument for research applications as well as for clinical evaluation. Its use is recommended for psychologists and mental health professionals in both diagnostic settings and empirical studies.

## 1 Introduction

In recent years, mental health has been increasingly recognized as a fundamental pillar of public health, as psychological disorders exert profound effects on quality of life, individual and social functioning, and societal productivity (World Health Organization, 2022). Among mental health–related constructs, worry, as a future-oriented and repetitive cognitive process, plays a central role in the development and maintenance of various anxiety disorders, particularly generalized anxiety disorder (Borkovec et al., 2004; Gori et al., 2023). In addition, growing evidence suggests that worry is also involved in the development and maintenance of other psychological conditions, including eating disorders (Palmieri et al., 2021; Palmieri et al., 2023), substance use disorders (Mansueto et al., 2024), difficulties in emotion regulation (Mansueto et al., 2022; Mansueto et al., 2024), and depression (Chelminski and Zimmerman, 2003; Papageorgiou, 2006). These findings highlight the transdiagnostic role of worry across a broad range of psychological disorders. Research conducted in Persian-speaking populations has similarly emphasized the role of repetitive negative thinking, emotional beliefs, emotion dysregulation, and perceived emotional invalidation in the development of psychological distress and emotional disorders (Rezaei et al., 2024; Rezaei et al., 2025a; Rezaei et al., 2025b), further supporting the relevance of examining worry-related processes within Dari/Farsi-speaking contexts.

Addressing worry is of heightened importance in countries such as Afghanistan, where decades of war, political instability, poverty, forced displacement, and structural and social constraints have exposed the population to chronic and pervasive stressors, substantially increasing the risk of persistent psychological problems (Afsharzada et al., 2025; Al Jowf et al., 2022; Miller and Rasmussen, 2010). Within this context, university students represent a particularly vulnerable and strategically important population, as they are exposed to a wide range of academic, economic, and social stressors that may contribute to elevated levels of worry and mental health difficulties. Empirical evidence indicates that high levels of worry among students are associated with poorer academic performance, impaired concentration, psychological burnout, and increased symptoms of anxiety and depression (Beiter et al., 2015; Naghavi et al., 2022). In settings where access to specialized mental health services is limited, the accurate and reliable assessment of worry plays a crucial role in the early identification of at-risk individuals and the development of effective preventive and therapeutic interventions. Moreover, exposure to chronic social and individual stressors, including violence, insecurity, loss, and uncertainty, has been consistently associated with excessive worry and difficulties in controlling future-oriented negative thoughts (Chiu et al., 2024; Ghasemzadehbarki et al., 2024; Gori et al., 2023). Given Afghanistan’s prolonged exposure to sociopolitical instability and traumatic experiences, examining worry within this cultural context is of particular importance. These considerations underscore the importance of scientifically examining worry and its assessment tools within the Afghan cultural context.

The Penn State Worry Questionnaire (PSWQ) is one of the most widely used and well-validated self-report instruments for assessing trait worry, originally developed by Meyer et al. (1990). By focusing on the frequency, intensity, and uncontrollability of worry, the PSWQ has demonstrated satisfactory discriminative validity in distinguishing pathological worry from general anxiety and related emotional symptoms (Brown et al., 1992; Fresco et al., 2003; Startup and Erickson, 2006). To date, the PSWQ has been translated and psychometrically validated in a wide range of languages and cultural contexts, including Persian, Turkish, Spanish, Portuguese, Dutch, Chinese, and French, with findings consistently supporting its stable factor structure and robust psychometric properties (Dehshiri et al., 2009; Gana et al., 2002; Jiménez-Ros et al., 2019; Nuevo et al., 2007; Vander Heiden et al., 2010; Yılmaz et al., 2008; Zhong et al., 2009). However, despite its extensive international use, no formal translation, cultural adaptation, or validation of the PSWQ has yet been reported within the Afghan population. Although Dari/Farsi shares important linguistic similarities with the Persian (Farsi) language spoken in Iran, substantial differences in sociocultural context, educational conditions, lived experiences, and patterns of psychological distress may influence how worry-related items are interpreted and endorsed among Afghan populations. Therefore, psychometric findings obtained in Iranian samples cannot be assumed to generalize directly to Afghan individuals. In addition, cultural and contextual differences may contribute to differential item functioning across Dari/Farsi-speaking populations, particularly for reverse-scored items or items reflecting culturally nuanced expressions of worry. This further underscores the importance of validating the PSWQ specifically within Afghan samples. Given the scarcity of standardized mental health assessment instruments in Afghanistan and the potential clinical relevance of worry among university students, validating the PSWQ within this population is of substantial importance. In light of these theoretical, clinical, and cultural considerations, the present study aimed to translate and validate the Dari/Farsi version of Penn State Worry Questionnaire (PSWQ) among Afghan university students. Based on previous international validation studies, it was hypothesized that the Dari/Farsi version of the PSWQ would demonstrate a unidimensional factor structure, satisfactory internal consistency and test–retest reliability, and significant positive correlations with depression, anxiety, and stress, thereby supporting its construct and criterion validity.

## 2 Methods

### 2.1 Methods and participants

It employed a descriptive and validation-based approach. Participants in this study were undergraduate students studying at Herat University, Afghanistan, during the 2024–2025 academic year. Prior to data collection, sample size was estimated using G*Power, assuming a moderate effect size, a statistical power of 0.90, and a significance level of 0.05. The analysis indicated that a minimum of 220 participants was required. To compensate for possible attrition and incomplete responses, a larger sample of 270 students was targeted.

Data were collected from a convenience sample of 270 students who completed an online questionnaire administered through the Qualtrics platform (Qualtrics, Provo, UT). Eligibility criteria included being between 18 and 45 years of age, being currently enrolled as an undergraduate student, and providing informed consent to participate in the study. Participants were excluded if they self-reported a history of psychiatric disorders, current psychiatric treatment, experience of parental loss or divorce, or if they submitted incomplete questionnaires. In addition, participants with elevated social desirability scores (>12 on the Marlowe-Crowne Social Desirability Scale) were excluded to minimize the risk of response bias and improve data validity. To assess and control for response bias, the Marlowe-Crowne Social Desirability Scale was administered. This widely used instrument comprises 33 dichotomous (true/false) items, with higher total scores reflecting a stronger tendency toward socially desirable responding. Consistent with theoretical considerations and prior empirical evidence, scores exceeding 12 were interpreted as indicating an elevated level of response distortion, potentially threatening data validity. Accordingly, sixteen participants were excluded from the final analysis due to incomplete questionnaire responses or social desirability scores exceeding the predetermined cutoff value.

After these exclusions, the final analytical sample consisted of 254 participants aged 18 to 45 years. The demographic profile of the sample is presented in Table 1.

**Table 1:** Demographic characteristics of the sample (*N* = 254).

Variable	M (SD)/*n* (%)
Age (years)	21.67 (3.23)
Gender	
Female	90 (35.40%)
Male	164 (64.6%)
Marital status	
Single	162 (63.80%)
Married	92 (36.20%)
Employment status	
Employed	119 (46.90%)
Unemployed	135 (53.10%)

M, mean; SD, standard deviation. The percentages are based on the total sample size.

Participants were also invited to voluntarily provide contact information if they agreed to take part in a second administration of the questionnaire. From those who consented, forty individuals were randomly selected to complete the PSWQ again after a two-week interval in order to evaluate test–retest reliability. All participants received comprehensive information about the study objectives and procedures and provided electronic informed consent prior to participation. Additionally, they were assured that involvement in the study posed no physical or psychological harm.

### 2.2 Procedure and material

Researchers employed a rigorous forward–backward translation procedure to adapt the PSWQ into the Dari/Farsi language. Initially, two members of the research team independently translated the questionnaire from English into Dari/Farsi, ensuring that cultural nuances and linguistic accuracy were carefully considered. The preliminary Dari/Farsi version was then created by comparing, reconciling, and refining the two translations. Subsequently, this version was back-translated into English by two professional translators who were blind to the original PSWQ to prevent bias and ensure conceptual consistency. The research team reviewed the original and back-translated versions in detail, identifying discrepancies and resolving them through multiple rounds of discussion. Finally, an expert panel evaluated the items for clarity, cultural appropriateness, and semantic equivalence, leading to full agreement on the finalized Dari/Farsi version of the questionnaire.

### 2.3 The Penn State Worry Questionnaire

The Penn State Worry Questionnaire (PSWQ) is a self-report questionnaire developed by Meyer et al. (1990) to assess worry (Meyer et al., 1990). The questionnaire has 16 items, which are scored on a 5-point Likert scale (*1 = Not at all typical, 2 = Rarely typical of me, 3 = Somewhat typical of me, 4 = Often typical of me, 5 = Very typical of me*). Five items of the PSWQ are considered negative (items 1, 3, 8, 10, and 11), which assess the absence or controllability of worry; the other eleven are positive (items 2, 4, 5, 6, 7, 9, 12, 13, 14, 15, and 16) and reflect the presence, frequency, and intensity of excessive and uncontrollable worry. The negative items are reversely coded when calculating the total score of the PSWQ. So, the total score of the PSWQ ranges from 16 to 80, with a higher score representing greater worry. As the PSWQ is not a diagnostic tool, there are no cut-off scores. The PSWQ has been found to have good construct and internal reliability in a sample of participants with GAD (Brown et al., 1992; Dear et al., 2011). Internal consistency for the combined sample was *α* = 0.97.

### 2.4 Depression Anxiety Stress Scale-21 items

DASS-21, developed by Lovibond and Lovibond (1995), is a self-report instrument designed to measure the emotional states of depression, anxiety, and stress. The instrument consists of 21 items organized into three equally sized subscales, each comprising seven items. Responses are scored using a four-point Likert-type scale ranging from 0 to 3, where higher values reflect greater symptom severity. Participants were instructed to rate each statement based on their experiences during the previous week. Subscale scores can range from 0 to 21, with higher scores indicating more pronounced symptom levels. The DASS-21 is applicable across a wide age range, from adolescents aged 14 to older adults, and its psychometric and clinimetric properties have been supported by numerous studies (Lovibond and Lovibond, 1995; Mansueto et al., 2023). In a recent validation study conducted among 1,318 Afghan participants, Neyazi et al. (2024) reported a Cronbach’s alpha coefficient of 0.94, indicating excellent internal consistency.

### 2.5 Statistical analysis

Statistical analyses were performed using SPSS version 27 and AMOS version 24. Due to sample size considerations, measurement invariance across gender was not examined in the present study.

To evaluate construct validity, a confirmatory factor analysis (CFA) based on a one-factor model was conducted to examine the latent structure of the PSWQ and its underlying construct. Criterion-related validity was assessed by computing Pearson correlation coefficients between PSWQ scores and the depression, anxiety, and stress subscales of the DASS-21. Reliability analyses included the estimation of internal consistency using Cronbach’s alpha coefficient, as well as temporal stability through test–retest reliability assessed with Pearson’s correlation.

## 3 Results

A total of 254 undergraduate students participated in the study by completing the online questionnaire. Descriptive statistics for each PSWQ item are presented in Table 2. The average item scores were generally situated within the lower to mid-range of the Likert scale, reflecting low to moderate levels of self-reported worry among participants. Item analysis further revealed strong item–total correlations, with coefficients ranging from 0.48 to 0.90, exceeding the recommended cutoff of 0.30 and indicating that all items contributed meaningfully to the measurement of the underlying construct. Notably, the reverse-coded items (Items 3 and 11) showed relatively higher mean scores compared to the remaining items. This pattern may reflect potential method effects or differences in participants’ interpretation of negatively worded items, a phenomenon previously reported in studies involving the PSWQ and other self-report measures containing reverse-scored items. Nevertheless, the item–total correlations for these items remained within acceptable ranges, suggesting that they still contributed meaningfully to the overall construct measurement.

**Table 2:** Descriptive statistics for PSWQ.

Items	Mean	SD	Skewness	Kurtosis	Item-total correlation
1	2.75	1.39	0.22	−1.22	0.55^**^
2	2.54	1.10	0.20	−0.69	0.58^**^
3	3.42	1.52	−0.38	−1.35	0.56^**^
4	2.44	1.34	0.39	−1.13	0.88^**^
5	2.50	1.26	0.39	−0.96	0.53^**^
6	2.48	1.26	0.48	−0.75	0.61^**^
7	2.48	1.44	0.47	−1.14	0.88^**^
8	3.02	1.48	0.01	−1.38	0.60^**^
9	2.14	1.20	0.83	−0.28	0.78^**^
10	2.25	1.24	0.74	−0.40	0.48^**^
11	3.39	1.49	−0.40	−1.30	0.53^**^
12	2.32	1.39	0.61	−0.94	0.87^**^
13	2.46	1.44	0.54	−1.10	0.90^**^
14	2.34	1.23	0.61	−0.55	0.54^**^
15	2.38	1.45	0.59	−1.05	0.89^**^
16	2.42	1.29	0.63	−0.65	0.55^**^
PSWQ	41.33	14.65	0.21	−1.18	–

** Correlation is significant at the 0.01 level (2-tailed).

Next, the construct validity was examined using confirmatory factor analysis.

### 3.1 Validity

**Figure 1:**
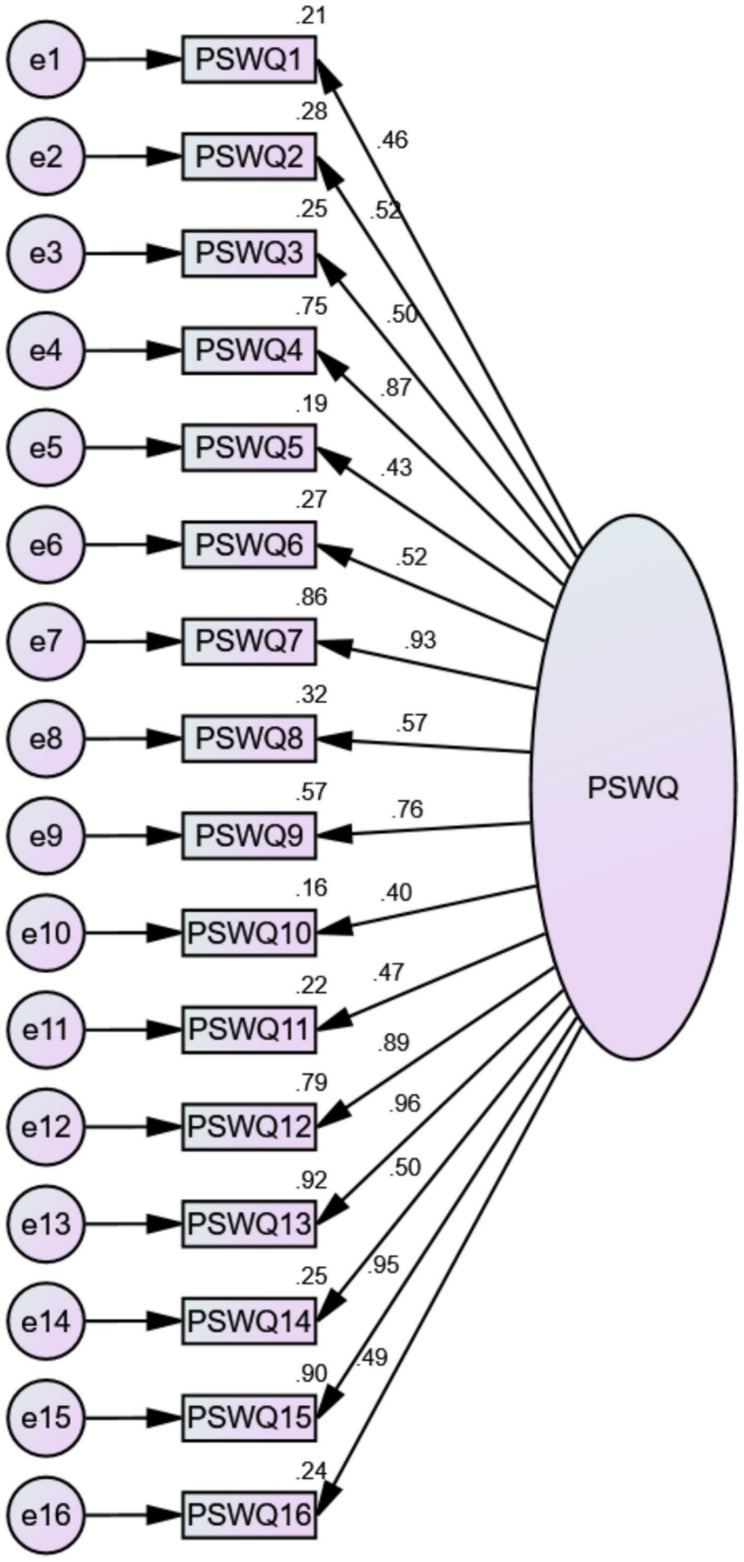
Confirmatory factor analysis for PSWQ.

Construct validity of the instrument was investigated through confirmatory factor analysis (CFA), with the aim of testing whether the observed data adequately supported the hypothesized factor structure. In the following, single-factor analyses are presented (Figure 1).

Analysis of the measurement model revealed that all PSWQ items loaded satisfactorily on the latent factor, with standardized factor loadings ranging from 0.40 to 0.95, exceeding the recommended minimum threshold. Although Item 10 demonstrated a comparatively lower factor loading (0.40) relative to the other items, it remained above the minimum acceptable threshold commonly reported in psychometric research and was therefore retained in the model. The goodness-of-fit indices for the measurement model, estimated using the maximum likelihood method, are reported in Table 3.

**Table 3:** Fit indices for the single-factor models.

Index	Single-factor model	Decision criterion
(X^2^)	269.06	*P* > 0.05
(Df)	104	–
P (Value)	0.01	*P* > 0.05
(X^2^/df)	2.58	CMIN/DF < 3
Comparative Fit Index (CFI)	0.94	CFI > 0.90
Goodness of Fit Index (GFI)	0.90	GFI > 0.90
Incremental Fit Index (IFI)	0.94	IFI > 0.90
root mean square error of approximation (RMSEA)	0.07	RMSEA <0.08

CFA results indicated that the single-factor model demonstrated an acceptable-to-good fit to the observed data (χ^2^/df = 2.58, CFI = 0.94, RMSEA = 0.07). Table 4 also provides the evidence for the instrument’s criterion validity.

**Table 4:** Criterion validity results.

Variables	Depression	Anxiety	Stress
PSWQ	0.53**	0.55**	0.59**

** Correlation is significant at the 0.01 level (2-tailed).

Evidence for criterion validity indicated that PSWQ scores were positively and significantly correlated with depression (*r* = 0.53), anxiety (*r* = 0.55), and stress (*r* = 0.59), all at the *p* < 0.05 level. These findings suggest that the PSWQ demonstrates acceptable criterion-related validity when applied to a sample of Afghan university students.

### 3.2 Reliability

The internal consistency of the PSWQ was examined using Cronbach’s alpha coefficient, which yielded a high value (*α* = 0.92). In addition, the stability of the instrument over time was supported by test–retest analysis conducted over a two-week interval, resulting in a correlation coefficient of *r* = 0.70.

## 4 Discussion

The primary objective of the present study was to translate and psychometrically validate the Penn State Worry Questionnaire (PSWQ) for use among Afghan university students. The translation procedure followed established forward–backward translation guidelines to ensure both linguistic accuracy and conceptual equivalence. After completing the translation process, a detailed psychometric assessment was carried out to evaluate the reliability and validity of the Dari/Farsi version of the PSWQ. Confirmatory factor analyses were employed to examine the underlying factor structure of the scale. In this study, one model was examined. The results showed that all items loaded significantly onto their respective factors, mirroring the structure proposed in the original scale developed by Meyer et al. (1990). Results from the confirmatory factor analysis provided general support for the proposed single-factor structure of the PSWQ, indicating that the model demonstrated an overall acceptable fit to the observed data. This finding is consistent with previous studies, as several investigations have supported the unidimensional (one-factor) structure of the (PSWQ), often noting the presence of method effects associated with reverse-scored items (Hochheimer et al., 2025; Meyer et al., 1990; Zhong et al., 2009). It should also be noted that Item 10 demonstrated a comparatively lower factor loading than the remaining items, which may suggest that this item is somewhat less strongly associated with the general worry construct in the present sample. Nevertheless, the loading remained within the minimally acceptable range and the item was retained to preserve consistency with the original PSWQ structure. Findings further indicated that the PSWQ possesses strong internal consistency and satisfactory test–retest reliability, suggesting that the instrument produces stable and reliable scores across time. However, several items (particularly items 4, 7, 12, 13, and 15) demonstrated very high item–total correlations, which may suggest a degree of conceptual overlap or redundancy among some PSWQ items. Although this pattern supports strong internal consistency, it may also indicate that certain items capture highly similar aspects of worry-related cognition. Future studies may benefit from further examining item functioning and potential redundancy using additional psychometric approaches such as item response theory or bifactor modeling.

Consistent with previous research (Bottesi and Spoto, 2025; Zlomke, 2009), the total scores of the PSWQ were positively correlated with levels of depression, anxiety, and stress, supporting the scales’ criterion validity. The observed associations between worry and symptoms of depression, anxiety, and stress are also clinically meaningful. Previous studies conducted in both clinical and non-clinical populations have similarly demonstrated that elevated levels of worry are strongly associated with greater psychological distress, affective symptoms, and emotional dysfunction (Chelminski and Zimmerman, 2003; Mansueto et al., 2023; Mansueto et al., 2022; Mansueto et al., 2024; Papageorgiou, 2006). These findings support the conceptualization of worry as a transdiagnostic cognitive process that contributes to the maintenance and exacerbation of multiple forms of psychological distress. Within the Afghan context, where university students are frequently exposed to academic pressure, financial hardship, uncertainty about the future, and broader sociopolitical stressors, excessive worry may represent an important psychological vulnerability factor associated with poorer emotional well-being and increased mental health difficulties. From a clinical and practical perspective, the availability of a valid and reliable Dari/Farsi version of the PSWQ may facilitate the early identification of students experiencing maladaptive and uncontrollable worry. The instrument may therefore be useful in university counseling centers, mental health screening programs, and psychological research aimed at identifying individuals at risk for anxiety-related and affective difficulties. Furthermore, assessing worry through the PSWQ may help clinicians and counselors develop more targeted interventions focused on cognitive processes such as repetitive negative thinking, intolerance of uncertainty, and emotion regulation difficulties. The reliability findings were also consistent with the original PSWQ development study (Meyer et al., 1990), further supporting the internal consistency and temporal stability of the Dari/Farsi version of the instrument.

Thus, the Dari/Farsi translation of the PSWQ demonstrates sufficient validity and reliability, with the findings generally aligning with previous validation studies conducted across different cultural contexts (Dehshiri et al., 2009; Gana et al., 2002; Jiménez-Ros et al., 2019; Nuevo et al., 2007; Vander Heiden et al., 2010; Yılmaz et al., 2008; Zhong et al., 2009). Based on the results obtained, the questionnaire exhibits sound psychometric qualities within the Afghan cultural context and, given its brief and straightforward format, can be employed by researchers and mental health professionals in academic and applied settings. Nevertheless, practical considerations such as variations in literacy levels, limited internet accessibility in some regions, differences between online and paper-based administration formats, and the potential influence of social desirability in face-to-face assessments should be carefully considered when implementing the instrument in broader Afghan contexts. As an effective instrument for assessing worry, it enables more accurate evaluations, facilitates timely actions, and supports improvements in clinical interventions within counseling and psychological settings. Despite these strengths, the present study has certain limitations that should be acknowledged. First, the study relied on a convenience-based, non-probabilistic sample drawn exclusively from students at Herat University, which may limit the generalizability of the findings to other Afghan student populations or broader community samples. Moreover, the study did not include a clinical sample, such as individuals diagnosed with generalized anxiety disorder or other anxiety-related conditions. Consequently, evidence regarding known-groups validity and the discriminative capacity of the PSWQ between clinical and non-clinical populations could not be evaluated. In addition, participants with elevated social desirability scores were excluded from the analyses in an effort to minimize response bias and improve data quality. Although this procedure may have strengthened the internal validity of the findings, it may also have reduced the representativeness of the sample and potentially contributed to more favorable psychometric estimates. In terms of measurement-related limitations, criterion validity was assessed solely using the DASS-21 due to the limited availability of validated psychological instruments in the Afghan context. Furthermore, discriminant validity was not examined because no theoretically unrelated constructs were included in the assessment battery. Another limitation is that measurement invariance across gender was not evaluated; therefore, it remains unclear whether the factor structure and psychometric properties of the Dari/Farsi PSWQ operate equivalently among male and female students. In addition, the present study examined only the original single-factor structure of the PSWQ. Given previous evidence suggesting potential method effects associated with reverse-scored items, future studies should investigate alternative structural models, including bifactor and method-factor approaches, to further clarify the dimensionality of the instrument within Afghan samples. In light of these limitations, several directions for future research are recommended. Future studies are encouraged to employ additional complementary measures alongside the PSWQ, such as the Generalized Anxiety Disorder Scale (GAD-7), the Perseverative Thinking Questionnaire (PTQ), the Ruminative Response Scale (RRS), and conceptually unrelated constructs to more comprehensively evaluate convergent and discriminant validity, ideally using multitrait–multimethod matrix designs. Future research should also examine the performance of the PSWQ in clinical populations and investigate its ability to differentiate between clinical and non-clinical groups. Additionally, expanding sample sizes, incorporating participants from diverse backgrounds, applying probabilistic sampling techniques, and integrating qualitative methods such as interviews may further strengthen the robustness and generalizability of future findings. Finally, future studies may benefit from examining the associations between PSWQ scores and related constructs, including perceived stress, psychological capital, emotion regulation, and rumination.

## 5 Conclusion

In conclusion, the results of this investigation offer compelling support for the psychometric adequacy of the Dari/Farsi adaptation of the Penn State Worry Questionnaire (PSWQ). When administered to a non-clinical group of Afghan university students, the instrument demonstrated characteristics consistent with a valid and reliable measure of worry. The scale demonstrated a clear and stable factor structure, along with satisfactory levels of internal consistency, confirming the coherence and reliability of its items. In addition, the retest reliability results supported the temporal stability of the PSWQ, indicating that the measure produces consistent scores over time.

The results of the confirmatory factor analyses revealed that the single-factor model demonstrated an overall acceptable fit to the observed data, supporting the theoretical structure of worry within the Afghan cultural context. Furthermore, the PSWQ showed acceptable criterion validity by effectively capturing the expected associations between worry and key indicators of psychological distress, including depression, anxiety, and stress. These associations further emphasize the potential clinical relevance of worry among Afghan university students and highlight the importance of early psychological assessment and intervention. Accordingly, the Dari/Farsi PSWQ may serve as a useful instrument for the assessment and monitoring of worry-related experiences among Afghan university students in academic and research settings. Taken together, these findings suggest that the Dari/Farsi version of the PSWQ is a reliable and culturally appropriate instrument for research and preliminary psychological assessment purposes among non-clinical Dari/Farsi-speaking Afghan populations. Nevertheless, additional research involving larger and clinical samples is needed to establish normative data and clinically meaningful cutoff scores for the PSWQ within Afghan populations.

## Data Availability

The original contributions presented in the study are included in the article/supplementary material, further inquiries can be directed to the corresponding author.
